# Estimating the cumulative risk of postnatal depressive symptoms: the role of insomnia symptoms across pregnancy

**DOI:** 10.1007/s00127-021-02101-0

**Published:** 2021-05-07

**Authors:** Johanna T. Pietikäinen, Tommi Härkänen, Päivi Polo-Kantola, Hasse Karlsson, Tiina Paunio, Linnea Karlsson, E. Juulia Paavonen

**Affiliations:** 1 Department of Psychiatry, Helsinki University Hospital and University of Helsinki, Helsinki, Finland; 2 Department of Public Health and Welfare, Finnish Institute for Health and Welfare, Mannerheimintie 166, P. O. Box 30, 00271 Helsinki, Finland; 3 Department of Obstetrics and Gynaecology, Turku University Hospital, Turku University, Turku, Finland; 4 Department of Pulmonary Diseases and Allergology, Sleep Research Centre, University of Turku, Turku, Finland; 5 Department of Clinical Medicine, The FinnBrain Birth Cohort Study, Turku Brain and Mind Center, University of Turku, Turku, Finland; 6 Department of Psychiatry, Turku University Hospital and University of Turku, Turku, Finland; 7 Centre for Population Health Research, Turku University Hospital and University of Turku, Turku, Finland; 8 Department of Psychiatry and SleepWell Research Program, University of Helsinki and Helsinki University Hospital, Helsinki, Finland; 9 Department of Child Psychiatry, Pediatric Research Center, Helsinki University Hospital and University of Helsinki, Helsinki, Finland

**Keywords:** Cohort studies, Insomnia, Prospective, Perinatal, Postpartum depression, Heat map

## Abstract

**Purpose:**

Insomnia symptoms during late pregnancy are a known risk for postnatal depressive symptoms (PDS). However, the cumulative effect of various risk factors throughout pregnancy has not been explored. Our aim was to test how various insomnia symptoms (sleep latency, duration, quality, frequent night awakenings, early morning awakenings) and other risk factors (e.g., history of depression, symptoms of depression and anxiety, as well as sociodemographic factors) in early, mid-, and late pregnancy predict PDS.

**Methods:**

Using data from the FinnBrain Birth Cohort Study and logistic regression analyses, we investigated the associations of distinct insomnia symptoms at gw 14, 24, and 34 with depressive symptoms (Edinburgh Postnatal Depression Scale score ≥ 11) 3 months postnatally. We also calculated separate and combined predictive models of PDS for each pregnancy time point and reported the odds ratios for each risk group.

**Results:**

Of the 2224 women included in the study, 7.1% scored EPDS ≥ 11 3 months postnatally. Our predictive models indicated that sleep latency of ≥ 20 min, anxiety in early pregnancy, and insufficient sleep during late pregnancy predicted the risk of PDS. Furthermore, we found highly elevated odds ratios in early, mid-, and late pregnancy for women with multiple PDS risk factors.

**Conclusion:**

Screening of long sleep latency and anxiety during early pregnancy, in addition to depression screening, could be advisable. Odds ratios of risk factor combinations demonstrate the magnitude of cumulating risk of PDS when multiple risk factors are present.

**Supplementary Information:**

The online version contains supplementary material available at 10.1007/s00127-021-02101-0.

## Introduction

Postpartum depression (PPD) affects approximately 8% of women in European countries [1], and up to 13–19% in other high-income countries [2]. PPD has potentially long-term adverse consequences for the mother, child, and entire family [3]. Several risk factors for PPD have been identified, including anxiety during pregnancy, multiparity, a low-income level, and a history of depression [4]. Insomnia symptoms during late pregnancy are one of these risk factors, as summarized in several reviews [5–7] and a recent meta-analysis [7]. Nevertheless, the effect and magnitude of increased risk with each cumulating factor have not been explored.

Perinatal psychiatric disorders are closely related to disturbances in sleep. According to a recent review [5], 42–100% of women with postnatal depression, anxiety or psychosis also suffered from concurrent insomnia. Prenatal insomnia symptoms of varying severity are very common; they are reported in at least 50% of women in different stages of pregnancy, depending on the characteristics of the study population and measurement methods [8]. Poor sleep during pregnancy has been associated with both maternal and foetal health risks, such as maternal hypertension and gestational diabetes, poor obstetric outcomes, foetal growth restriction, and the risk of preterm delivery, as well as interpersonal distress and mood disorders [9, 10].

Most of the studies addressing the connection between sleep during pregnancy and postnatal depression have been cross-sectional [11], have had a low number of participants [12], have focused on insomnia symptoms during late pregnancy [13, 14] versus depressive symptoms in the postnatal period, have had a high drop-out rate (for example, 75% from the baseline) [15], or have not differentiated prenatal sleeping problems between the pregnancy trimesters [16]. Those studies that have investigated insomnia symptoms during the first and second pregnancy trimesters [17] have not compared the role of specific early insomnia symptoms in predicting postnatal depressive symptoms.

Why should we be interested in investigating maternal insomnia symptoms that already begin during the early stages of pregnancy? First, maternal insomnia symptoms are highly prevalent, even in the first months of pregnancy, and sleep quality typically continues to deteriorate as pregnancy progresses [18]. Insomnia symptoms seem to persist until the postnatal period, especially in first-time mothers [19, 20]. Second, maternal sleep and depression are connected to sleep in the offspring [21]. Although insomnia is a modifiable and treatable risk factor for PPD, surprisingly little is known about insomnia symptoms experienced during early and mid-pregnancy, and particularly whether they are associated with postnatal depression. Treatment of insomnia could prevent maternal psychopathology and associated adverse consequences for the mother and child. Moreover, earlier identification of mothers at risk could allow more time for interventions during pregnancy.

Here, we investigated whether different insomnia symptoms from early pregnancy onwards predict increased postnatal depressive symptom (PDS) levels, using a large population-based sample of over 2000 women. Although depression in pregnancy predicts postnatal depression, not all mothers with postnatal depression have prenatal depressive symptoms. Moreover, not all women with insomnia symptoms during pregnancy are depressed, despite insomnia being one of the symptoms of depression. We constructed predictive models separately for early, mid-, and late pregnancy symptom profiles to better understand which insomnia symptoms, in combination with other known risk factors, at different time-points during pregnancy are the most predictive of PDS. We hypothesized that postnatal depressive symptoms can be predicted by prenatal risk factors such as anxiety during pregnancy, multiparity, and history of depression, and that adding sleep parameters would enhance the model fit. Furthermore, we hypothesized that insomnia symptoms already occurring during early and mid-pregnancy, and especially the accumulation of several insomnia symptoms and other risk factors, would increase the risk of PDS.

## Methods

### Participants

This study was part of the Finnish FinnBrain Birth Cohort Study (www.finnbrain.fi) recruited between December 2011 and April 2015 from three maternity welfare clinics in the South Western Hospital District and Åland Islands area in Finland. The study recruitment and protocol are described in detail elsewhere [22]. The parent study arm in the cohort is a prospective observational study focusing on how parental stress, health, and other characteristics during the prenatal and early life postnatal periods influence offspring health and brain development. Parental health and factors influencing these trajectories are also being investigated independently from child outcomes. The main domains of interest in this arm are parental mood, anxiety disorders, and sleep. All procedures involving human subjects were approved by the Ethics Committee of the South Western Hospital District (ETMK 57/180/2011, 14.6.2011 § 168). Written informed consent was obtained from all subjects. In total, 3808 women (66% of the total) informed about the study chose to participate. The questionnaire data for this study were collected at gestational week (gw) 14 (early pregnancy, time point T1; mean = 14.98 weeks, SD 1.31), gw 24 (mid-pregnancy, time point T2; mean = 25.31 weeks, SD 1.48), gw 34 (late pregnancy, time point T3; mean = 35.08 weeks, SD 1.03), and 3 months postpartum (time point T4; mean = 104.14 days after delivery, SD 9.47). The response rates were 100% (*n* = 3095) at T1, 90.0% (*n* = 2784) at T2, 84.3% (*n* = 2609) at T3 and 73.0% (*n* = 2260) at T4. The women who responded at 3 months postpartum and within accepted time ranges were eligible for this study, leaving a final sample of 2118 mothers at T1, 2152 at T2, 2095 at T3, and 2224 at T4. Complete data were obtained from all time-points for 1504 women.

### Measures

The data for this study were based on registry data and self-report questionnaires. Marital status, age, parity, educational level, income level, smoking during pregnancy, somatic diseases/disorders, and history of depression were inquired in the T1 questionnaires. The use of antidepressant and sleep medications was inquired repeatedly. Age was used as a continuous variable, whereas parity was dichotomized as primi- or multipara; educational level was categorized as 0 = mid/low (up to 12 years of education), 1 = high/vocational (college level or university of applied sciences, 12–15 years of education), and 2 = university degree (over 15 years of education); income level was categorized as ≤ €1000, €1001–2000 and > €2000 per month; smoking was dichotomized as no or yes if the mother had reported smoking in either questionnaires or registries and somatic diseases/disorders (excluding allergies) were dichotomized as no or yes. Missing values were replaced by an individual mean in the corresponding scale when no more than 33% of answers in the scale were missing.

The Edinburgh Postnatal Depression scale (EPDS) [23] is a ten-item four-point scale measuring cognitive and affective symptoms of depression during the previous 7 days. Seven of the items on the scale are reverse-scored, and a total score is then calculated (range 0–30). We used the cut-off level of ≥ 11 (approximately 90th percentile) to indicate an increased level of depressive symptoms. Several different EPDS cut-off points have been used, and as Matthey et al. [24] pointed out, cut-offs established for English-speaking populations might not be applicable to other populations. The EPDS has not been validated in the Finnish population. Depressive symptoms were measured at T1, T2, T3,, and T4.

The anxiety subscale of Symptom Checklist 90 (SCL-90) [25, 26] was used to measure women’s prenatal anxiety at T1, T2 and T3. The scale consists of ten items rated on a five-point Likert scale. The scores were summed (range 0–40), and we used a cut-off level of ≥ 10 (22) to indicate increased anxiety.

The Basic Nordic Sleep Questionnaire (BNSQ) is a self-report scale of sleep during the previous month [27]. The questions measure sleep duration and quality, sleep latency, and the number of night awakenings per night and of too early morning awakenings per week. The use of sleep medication was also assessed. Most of the questions are rated on a five-point scale (1 = ‘never or less than once per month’, 2 = ‘less than once per week’, 3 = ‘on 1–2 days per week’, 4 = ‘on 3–5 days per week’, 5 = ‘daily or almost daily’). Sleep quality is rated as 1 = ‘well’, 2 = ‘rather well’, 3 = ‘neither well nor badly’, 4 = ‘rather badly’, or 5 = ‘badly’. To indicate clinically significant problems, the items were dichotomized as ‘one to two times per week or less’ or ‘three times per week or more’; the continuous sleep variables were also dichotomized to indicate sleep latency of ≥ 20 min and sleep duration of ≥ 6 and ≥ 7 h. The items of the scale were used separately to gather information on the prevalence of various insomnia symptoms. Thus, we did not use a summary score for the BNSQ.

The Athens Insomnia Scale (AIS) is a self-report questionnaire for sleep disturbances based on ICD-10 criteria [28]. In this study, we used three items concerning sufficient total sleep time, the sense of well-being, and physical and mental functioning during the day related to sleep. A question concerning the amount of sleep was dichotomized as 0 and 1 (= ‘sufficient’ and ‘slightly insufficient’) or 2 and 3 (= ‘markedly insufficient’ and ‘very insufficient or did not sleep at all’), whereas the other two items were dichotomized as follows: 0 and 1 (= ‘normal’ and ‘slightly decreased’) or 2 and 3 (= ‘markedly decreased’ and ‘very decreased’). At all time-points, we used these three items from the AIS questionnaire.

### Statistical analyses

Our aim was to investigate the risk of PDS, and we therefore selected logistic regression models as the primary statistical method rather than linear regression models. Logistic models also enabled us to construct cumulative risk estimates that could be visualized in heat maps, which is not possible with linear regression. However, as linear regression models have been commonly applied in analyzing the risk of PDS, we also performed sensitivity analyses using corresponding linear regression models to confirm the stability of the *p* values and therefore the conclusions (Online Resource 3).

The following dichotomized sleep variables (representing the different manifestations of insomnia symptoms) in T1–T3 served as the explanatory variables: sleep latency ≥ 20 min, night awakenings ≥ 3×/night, early morning awakenings ≥ 3×/week, ‘poor/rather poor’ sleep quality, short sleep ≤ 6 and ≤ 7 h, insufficient total sleep time, decreased well-being, and functional capability during the day.

To assess whether insomnia symptoms during pregnancy increased the risk of postnatal depressive symptoms (EPDS score ≥ 11 at T4), simple logistic regression analyses (Online Resource 1) were first conducted separately at the three pregnancy time-points (T1–T3) and for each sleep variable to identify significant predictive factors to be used in the next stage of statistical modelling. The models were adjusted for known confounding factors: mother’s age, parity (primi- vs multipara), educational level (three classes), income level (three classes), somatic illnesses/conditions,concurrent depressive symptoms (EPDS ≥ 11 at each pregnancy time point T1–T3), concurrent anxiety (SCL ≥ 10), and history of depression. These analyses were conducted with SPSS (version 25).

Next, we constructed a second series of logistic models (predictive models) to predict the risk of postnatal depressive symptoms (EPDS ≥ 11 at T4). First, we constructed the best predictive models for each time point (T1, T2, and T3) (Table 2). The explanatory factors in these models were the insomnia symptoms that were associated with PDS in the simple logistic regression models, as well as depression (EPDS score ≥ 11) and anxiety (SCL ≥ 10) at the concurrent pregnancy time point. At pregnancy time point T1, the background factors (mother’s age, parity, education, income, and history of depression) were also included in the model. To select the best model with the least number of variables, we used the complete case data of participants who had answered all items used in the predictive model analysis (*n* = 1504). We selected the best model by assessing the best predictive value and the smallest Akaike information criterion (AIC), so that the variables were removed from the model until no further reduction could be achieved [29]. We also investigated how well the models were able to predict the risk of PDS using McFadden and Nagelkerke [30, 31].

Next, we constructed cumulative models combining the best predictive models for each time point to explore the extent to which the follow-up data improved the model fit and its predictive value compared to the original model at time point T1. We combined the predictors of the three best-fitting models from time-points T1, T2, and T3, constructing combined models T1 + T2 and T1 + T2 + T3. This was intended to mimic the clinic situation where, at the first appointment, only the background variables and measured variables for T1 would be available; thereafter, the measured information would increase throughout the pregnancy (Table 2).

**Fig. 1:**
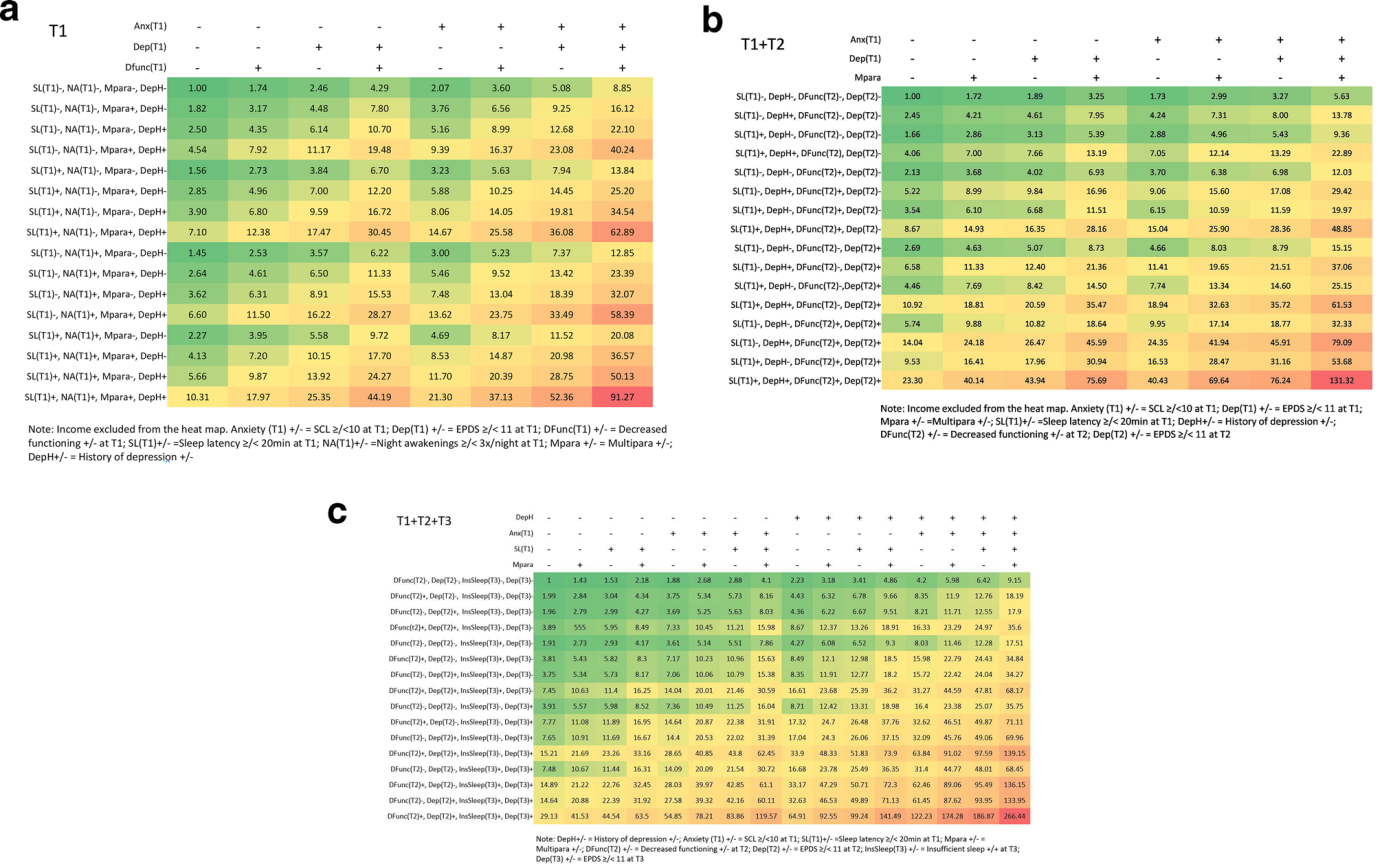
Heat maps to predict the risk of postnatal depressive symptoms 3 months postnatally. Values represent the odds ratio (OR) in relation to the lowest risk group (OR = 1). **a** Heat map in early pregnancy (background variables + T1 measures). **b** Heat map in middle pregnancy (background variables + T1 + T2 measures). **c** Heat map in late pregnancy (background variables + measures from T1 + T2 + T3)

Finally, to demonstrate the contrasts in various risk groups relative to the lowest risk category, we calculated the respective odds ratio (OR) estimates and summarized the results in three separate heat maps representing the cumulative risk at T1 (Fig. 1a), T1 + T2 (Fig. 1b) and T1 + T2 + T3 (Fig. 1c) for postnatal depressive symptoms at T4 (EPDS ≥ 11). In the first heat map (T1), we report significant background variables (parity, history of depression), as well as measures from T1; in the second heat map (T1 + T2), we added measures from T2; in the third (T1 + T2 + T3), we used all available information (background information and measures from T1 + T2 + T3). We excluded income from heat maps T1 and T1 + T2. The statistical software tool R (version 3.6.1, DescTools package) was used in all the predictive model analyses.

We performed attrition analysis of women who participated at T4 and at least once at T1, T2, or T3 (*n* = 2224) versus women who had dropped out (*n* = 1004). Women who had more depressive symptoms (EPDS ≥ 10), more anxiety (SCL > 10), and a lower educational and income level at T1 were more likely to drop out (*p* < 0.05), whereas there was no difference between the groups concerning history of depression. Furthermore, more women who had sleep onset difficulties (8.4% vs 5.2%, *p* < 0.001), woke up ≥ 3/night (16.7% vs 12.2%, *p* < 0.001), or had poor sleep quality (18.7% vs 12.2%, *p* < 0.001) at T1 dropped out of the study before T4.

As a sensitivity analysis, we repeated the analyses with the EPDS sum score without the sleep item “I have been so unhappy that I have had difficulty sleeping” (Online Resource 2).

## Results

The main background characteristics of the participants are described in Table 1. The mean EPDS score decreased slightly from T3 to T4. Approximately 3% of women reported the use of antidepressant medication (SSRI or SNRI) during pregnancy, and very few (0.5–0.9%) women used sleep medications more than once a month. Seven percent (*n* = 157) of the participants scored EPDS ≥ 11 at 3 months postnatally (T4). Descriptive data concerning changes in sleep are reported in detail elsewhere (Online Resource 4) [32].

**Table 1:** Characteristics of the sample

Measure	Criterion	Mean (SD)/% (*n*)
Participants	Gestational week 14	2118
	Gestational week 24	2152
	Gestational week 34	2095
	Postpartum (3 months)	2224
EPDS	Gestational week 14	4.98 (3.91)
	Gestational week 24	4.74 (3.99)
	Gestational week 34	4.72 (4.00)
	Postpartum (3 months)	4.25 (3.82)
EPDS ≥ 11	Gestational week 14	8.9%
	Gestational week 24	9.2%
	Gestational week 34	9.3%
	Postpartum (3 months)	7.1%
SCL	Gestational week 14	3.13 (3.82)
	Gestational week 24	3.74 (4.21)
	Gestational week 34	3.09 (3.82)
	Postpartum (3 months)	2.58 (3.57)
SCL ≥ 10	Gestational week 14	6.6%
	Gestational week 24	9.1%
	Gestational week 34	7.1%
	Postpartum (3 months)	5.8%
Mother’s age	At delivery	30.63 (4.47)
Marital status	Married	55.5% (1235)
	Non-married	42.0% (934)
	Divorced	0.9% (20)
	Registered relationship	0.2% (5)
Lives with a partner	Gestational week 14	93.9% (2085)
	Gestational week 34	97.2% (2153)
Previous deliveries	None	52.7% (1171)
	At least one	47.2% (1051)
Education	Mid/low	34.0% (719)
	High/voc	30.0% (634)
	High	35.9% (759)
Maternal monthly income (T1)	≤ 1000 €	20.1% (446)
	1001–2000 €	50.3% (1061)
	≥ 2000 €	28.6% (604)
Smoking during pregnancy (registry + questionnaire)	Early pregnancy	8.5% (190)
	Late pregnancy	5.3% (118)
Somatic disease/disability	Yes	25.8% (573)
Mental (life-time) illness, self-report	Depression	12.4% (261)
Antidep. medication (SSRI/SNRI)	Gestational week 14	3.1% (64)
	Gestational week 34	2.9% (61)
Sleep medication 1×/month or more	Gestational week 14	0.8% (17)
	Gestational week 24	0.5% (11)
	Gestational week 34	0.9% (18)

*EPDS* the Edinburgh Postnatal Depression scale, *SCL* the anxiety subscale of Symptom Checklist 90, *SSRI* selective serotonin reuptake inhibitor, *SNRI* serotonin–norepinephrine reuptake inhibitor

### Simple logistic regression analyses

Each insomnia symptom and time point (T1–T3) was studied separately to evaluate the risk of PDS. These results are presented in Online Resource 1. At T1, the following insomnia symptoms were related to T4 depressive symptoms after adjustments: sleep latency ≥ 20 min (AOR 1.87, 95% CI 1.29–2.71), night awakenings ≥ 3×/night (1.86, 95% CI 1.20–2.87), an insufficient total sleep time (AOR 1.86, 95% CI 1.13–3.06), decreased well-being (1.93, 95% CI 1.21–3.09), and a decreased capability to function (1.91, 95% CI 1.17–3.17). At T2, only insufficient total sleep time (AOR 1.99, 95% CI 1.21–3.28), decreased well-being (AOR 2.23, 95% CI 1.31–3.79), and a decreased functional capability (AOR 2.02, 95% CI 1.24–3.29) remained significant. At T3, sleep latency ≥ 20 min (AOR 1.73, 95% CI 1.18–2.55), poor sleep quality (AOR 2.15, 95% CI 1.45–3.18), short sleep ≤ 7 h (AOR 1.74, 95% CI 1.18–2.57), and an insufficient total sleep time (AOR 2.76, 95% CI 1.73–4.40) predicted depressive symptoms at T4.

### Predictive models

To investigate how insomnia symptoms at T1–T3 predicted the risk of postnatal depressive symptoms after taking into account the selected background variables and measurement results from each time point, we constructed predictive models for each time point separately, as well as cumulative models (Table 2). In the first predictive model at T1, we used all available information at that time, as in a real-life situation: background factors and insomnia symptoms, EPDS and SCL measured at T1. The AIC for the best model at T1 was 675.55 and the best explanatory risk factors were multiparity, a low-income level, history of depression, sleep latency ≥ 20 min, night awakenings ≥ 3×/night, a decreased functional capability, increased anxiety, and EPDS ≥ 11. At T2, the best explanatory variables were decreased functioning, increased anxiety, and EPDS ≥ 11. At T3, the best explanatory risk factors were insufficient sleep, increased anxiety, and EPDS ≥ 11. To avoid multicollinearity issues, the background variables were only included in the T1 model and cumulative models.

**Table 2:** Predictive model for PDS (EPDS ≥ 11 at 3 months postpartum), *n* = 1504 (complete case data). (a) Best models for time-points T2 and T3. (b) Best reduced model for T1, combined model T1 + T2 (background variables + T1 and T2 measure), and T1 + T2 + T3 (all available information)

(a)	T2 best model			T3 best model		
Variable	Beta (SE)	*p*	AIC/BIC	Beta (SE)	*p*	AIC/BIC
Intercept	− 4.284 (0.341)	2E^−16^	689.682/710.945	− 4.969 (0.339)	2E^−16^	665.207/686.471
Decreased func. at T2	0.941 (0.271)	< 0.001		–	–	
Anxiety SCL ≥ 10 T2	0.671 (0.291)	0.021		–	–	
EPDS ≥ 11 T2	1.211 (0.286)	< 0.001		–	–	
Insuff. sleep T3	–	–		0.930 (0.255)	< 0.001	
Anxiety SCL ≥ 10 T3	–	–		0.478 (0.314)	0.127	
EPDS ≥ 11 T3	–	–		1.772 (0.265)	< 0.001	

(b)	T1 best model			T1 + T2 best model			T1 + T2 + T3 best model		
Variable	Beta (SE)	*p*	AIC/BIC	Beta (SE)	*p*	AIC/BIC	Beta (SE)	*p*	AIC/BIC
Intercept	− 5.237 (0.537)	< 2E^−16^	675.549/728.708	− 6.023 (0.575)	< 2E^−16^	660.136/713.294	− 6.231 (0.515)	< 2E^−16^	633.878/681.721
Parity (primi vs multi)	0.677 (0.226)	0.003		0.605 (0.228)	0.008		0.355 (0.237)	0.134	
Income level 1	0.495 (0.281)	0.080		0.398 (0.286	0.164		–	–	
Income level 2	− 0.085 (0.341)	0.804		− 0.091 (0.344)	0.790		–	–	
History of depression	0.937 (0.257)	< 0.001		0.906 (0.257)	< 0.001		0.801 (0.265)	0.003	
Sleep latency ≥ 20 min T1	0.440 (0.235)	0.062		0.504 (0.235)	0.032		0.425 (0.239)	0.075	
Night awakenings ≥ 3×/night T1	0.423 (0.276)	0.126		–	–		–	–	
Decreased funct. T1	0.603 (0.296)	0.042		–	–		–	–	
Anxiety SCL ≥ 10 T1	0.775 (0.340)	0.023		0.576 (0.341)	0.090		0.633 (0.314)	0.044	
EPDS ≥ 11 T1	0.851 (0.311)	0.006		0.649 (0.320)	0.043		–	–	
Decreased funct. T2	–	–		0.773 (0.279)	0.006		0.688 (0.286)	0.016	
EPDS ≥ 11 T2	–	–		0.938 (0.284)	< 0.001		0.671 (0.292)	0.021	
Insuff. sleep T3	–	–		–	–		0.650 (0.275)	0.018	
EPDS ≥ 11 T3	–	–		–	–		1.363 (0.270)	< 0.001	

*EPDS* the Edinburgh Postnatal Depression scale, *SCL* the anxiety subscale of symptom checklist 90

In the best cumulative model (T1 + T2 + T3), AIC was 633.88 and the variables best explaining postnatal depressive symptoms (T4) were multiparity, history of depression, sleep latency ≥ 20 min at T1, anxiety at T1, a decreased functional capability at T2, insufficient sleep at T3, and EPDS ≥ 11 at T2 and T3.

### Odds ratios for postnatal depressive symptoms (T4) related to combinations of risk factors in early, middle, and late pregnancy

ORs related to various combinations of explanatory factors were extracted separately for each time point. In models T1 and T1 + T2, we used all other risk factors except income level in the heat maps. We found that in early pregnancy (T1; Table 2; Fig. 1a), the risk of PDS was markedly increased in women with three or more risk factors compared to women with none. For example, in our sample, an OR of 91 was found for women (*n* = 3) with all seven risk factors (multiparity, history of depression, sleep latency ≥ 20 min, night awakenings ≥ 3×/night, decreased functional capability, increased depressive symptoms, and anxiety at T1) when compared to women without any. Model T1 was able to explain approximately 14.9% of increased depressive symptoms at T4 (McFadden 0.121, Nagerkerke 0.149). Similarly, the ORs for PDS using measurements from both early and mid-pregnancy (T1 + T2; Table 2; Fig. 1b) were highly elevated in women with all eight risk factors (multiparity, history of depression, low income, sleep latency ≥ 20 min at T1, increased depressive and anxiety symptoms at T1, decreased functional capability at T2, and increased depressive symptoms at T2) (OR 141; McFadden 0.142, Nagelkerke 0.174) when compared to women with none. In late pregnancy, the ORs for PDS at T4 were calculated after accounting for all of the cumulative significant information (background information as well as measures from T1, T2 and T3) (Fig. 1c). These models further confirmed that women with multiple risk factors had a highly elevated risk compared to women with no risk factors, for example, a 266-fold higher OR for women with all risks (multiparity, history of depression, sleep latency ≥ 20 min at T1, increased anxiety at T1, decreased functional capability at T2, increased depressive symptoms at T2, insufficient sleep at T3, and increased depressive symptoms at T3; *n* = 1; McFadden 0.174, Nagelkerke 0.212).

In sensitivity analysis, we repeated the predictive model analyses after removing the sleep item “I have been so unhappy that I have had difficulty sleeping” from the EPDS sum score. The main results remained the same (Online Resource 2). The model calculated without the sleep item was able to predict PDS as well as the other models. For example, the Nagelkerke for the T1 + T2 + T3 model was 0.213.

In a second sensitivity analysis, we compared linear (Online Resource 3) and logistic (Online Resource 1) regression models using EPDS as a continuous/dichotomized dependent variable and dichotomized insomnia symptom variables as the dependent variables. The following variables were significantly associated with postnatal depressive symptoms only in linear regression models: night awakenings > 3×/night and poor sleep quality in early pregnancy, sleep latency, sleep quality, and short sleep < 7 h in middle pregnancy, and early morning awakenings, decreased well-being, and decreased functioning in late pregnancy. All the other *p* values remained unchanged.

## Discussion

### Main findings and comparison with other studies

Our study had two aims: to study the association of maternal insomnia symptoms during pregnancy with postnatal depressive symptoms (PDS) and to create a cumulative model of risk factors for PDS. We demonstrated that not only insomnia symptoms in late pregnancy, but also those in early pregnancy relate to PDS, even after adjusting for concurrent depressive symptoms. To the best of our knowledge, the latter finding has not been reported before, hence emphasizing the importance of already evaluating sleep from the beginning of pregnancy. When we investigated the accumulation of risk by calculating the odds ratios for early, mid-, and late pregnancy and for distinct risk factor combinations, women with multiple risk factors had a markedly increased risk of PDS.

Our findings concerning insomnia symptoms during late pregnancy versus depressive symptoms 3 months postnatally are in agreement with previous studies [7, 33]. However, due to the scarcity of literature, comparison of our findings concerning early and mid-pregnancy is more difficult. A recent cross-sectional study conducted during the late second trimester and third trimester of pregnancy showed that sleep onset difficulties together with high nocturnal rumination were highly correlated with concurrent depressive symptoms and even suicidal ideation [34]. In non-pregnant samples, prolonged sleep latency has been shown to be a robust predictor of risk for depression: for example, Blanken et al. reported difficulty initiating sleep as a primary target for the prevention of depression in a study with a 6-year follow-up [35]. Finally, Kalmbach et al. found that during a 2-year follow-up, poor sleepers with difficulty initiating and maintaining sleep and having cognitive distortions concerning stressful events were particularly susceptible to developing depression [36]. However, the finding that long sleep latency already experienced during early pregnancy was associated with postnatal depressive symptoms is novel. Another novel finding is that in contrast to other pregnancy time-points, the predictive models at mid-pregnancy demonstrated that an insufficient total sleep time predicted postnatal depressive symptoms. Sleep changes as pregnancy progresses [37], and thus, both the screening time and screening items are essential for recognizing women at increased risk of PDS.

Although sleep problems are a diagnostic criterion in depression, sleep disruption has been shown to precede depression in non-pregnant populations [38–40], with the latest meta-estimate of OR 2.8 (95% CI 1.6–5.2) [40]. In a study by Ohayon and Roth [41], 40% of depressed patients reported insomnia before the first depressive episode, and 56% had insomnia preceding depression relapse. Hyperarousal of both sleep- and wake-promoting brain areas is considered as a central element of insomnia [42], which may represent inadequate resolution of emotional distress during rapid-eye-movement sleep [43]. In addition, sleep disruption can influence mood via systemic inflammation, oxidative stress, circadian rhythm disruption, altered melatonin secretion, and genetic factors (reviewed by Refs. [9, 44, 45]).

### Odds ratios

Risk calculators have been used for years in somatic medicine and clinical practice. However, to the best of our knowledge, there are no published reports using heat maps to demonstrate the risk of postnatal depressive symptoms. Most of risk factors used in our models (such as multiparity, history of depression, educational and income level, anxiety, and depression during pregnancy) have been reported in the previous literature [4]. As expected, depressive symptoms during pregnancy strongly predicted postnatal depressive symptoms. Furthermore, a decreased functional capability in pregnancy was also a risk factor. Importantly, sleep patterns in different phases of pregnancy, especially in early and mid-pregnancy, have generally been ignored in predictive models. Notably, our models revealed that one or two simultaneous risk factors only slightly increased the risk, depending on the factor, whereas the accumulation of three or more risk factors increased the risk substantially. In particular, the magnitude of relative risk accumulated with multiple risk factors.

Meaney et al. [46] have highlighted the importance of subclinical maternal depressive symptoms and, respectively, we have previously shown in another Finnish birth cohort that even mild maternal perinatal depressive symptoms are associated with children’s emotional problems at the ages of 2 and 5 years [47]. Thus, after careful consideration, we chose to use an EPDS cut-off score of 11 or more to indicate postnatal depressive symptoms. A cut-off of 13 or more is probably too high for the Finnish population, as in the FB cohort, only 3.8% of women had a score exceeding this.

Depressive symptoms can change during pregnancy, as reported by Korja et al. [48]. However, here, we focused on predicting postnatal depressive symptoms and thus adjusted the analyses for depressive symptoms during pregnancy.

It is noteworthy that while our predictive models were aimed at using background information and sleep to predict PDS, not all potential known risk factors were accounted for, and hence, these models are not fully comprehensive. In the future, factors such as marital satisfaction, the relationship with one’s own attachment figures, and the life-time history of trauma or abuse could be included in the risk analysis. The Antenatal Risk Questionnaire [49] or a similar scale could aid in evaluating such psychosocial risks. Nevertheless, our variables were easy to measure and provided important insights into the factors that contribute to sleep disturbances during pregnancy.

### Strengths and limitations

The strengths of this study were the normative population, the relatively large sample size, and the longitudinal study design with an acceptable drop-out rate when compared to other studies. Importantly, our study design considered clinical implications, which was also a major strength.

One limitation is the attrition in our study. When the women who had returned the questionnaire at 3 months postpartum were compared to those who did not return it, we found attrition to be higher among those women who had higher depressive symptoms in the early pregnancy. Moreover, significantly more women who at T1 had sleep onset difficulties (*p* < 0.001), woke up > 3×/night (*p* < 0.001), or had poor sleep quality (*p* < 0.001) dropped out of the study before T4. Thus, our results might not be generalized to the whole population, as the attrition was higher among women at increased risk. However, the detected associations could have been even stronger if these women had continued to participate in this study. In the future, objective sleep registration could provide complementary information. Another limitation was that EPDS has an item concerning sleep. However, our sensitivity analysis (Online Resource 2) repeated the analyses using the EPDS scale without the sleep item and confirmed that the models were able to predict PDS similarly to the original models.

## Conclusion

The use of heat map visualizations in clinical practice would help to identify women at increased risk of postnatal depressive symptoms. Of the risk factors included in the model, depression, anxiety, and insomnia symptoms during pregnancy are modifiable and treatable. However, neither anxiety nor insomnia symptoms are routinely screened at antenatal visits. Our results indicate that in addition to EPDS, both long sleep latency and anxiety should already be screened in early pregnancy to increase the sensitivity of screening for PDS. At the first antenatal visit, our early visual PDS odds ratio heat map could serve as a novel tool for identifying women with multiple risks, and help to evaluate the need for potential preventive interventions. For instance, cognitive behavioural therapy for insomnia (CBT-I) has also been shown to be a promising treatment option for pregnant women, whether delivered in person [50] or in digital format [51, 52].

## Supplementary Information

Below is the link to the electronic supplementary material.

Supplementary file1 (DOCX 34 KB)

Supplementary file2 (DOCX 27 KB)

Supplementary file3 (DOCX 36 KB)

Supplementary file4 (DOCX 16 KB)

## Data Availability

Finnish national legislation precludes sharing these data in open repositories. Access to pseudonymized data can be requested via research collaboration. Such requests can be directed to the Principal Investigators of the FinnBrain Birth Cohort Study (emails: hasse.karlsson@utu.fi and linnea.karlsson@utu.fi).
