# Supplementary material for: Estimating the cumulative risk of postnatal depressive symptoms: the role of insomnia symptoms across pregnancy
Source: Soc Psychiatry Psychiatr Epidemiol. 2021 May 7;56(12):2251–61. doi: 10.1007/s00127-021-02101-0 (PMC8558280; doi:10.1007/s00127-021-02101-0)
Supplement: Supplementary file 1 — Supplementary file1 (DOCX 34 KB) [file 127_2021_2101_MOESM1_ESM.docx]

**Online Resource 1.** Logistic regression analyses, EPDS ≥ 11 points three months postnatally as the dependent variable. The PDS+ column indicates the number of women with/without sleeping problem who had increased depressive symptoms (EPDS ≥ 11 at three months postpartum). All data are included (n = 2224).

1. Insomnia symptoms in early pregnancy (gw 14, T1) vs EPDS ≥ 11 three months postnatally

|  |  |  | T1 |  |  |  |  |  |  |  |  |  |
| --- | --- | --- | --- | --- | --- | --- | --- | --- | --- | --- | --- | --- |
| Sleep variables at T1 |  | PDS+  3 months | Unadj. |  |  | Adj. 1 | Adj. 2 |  |  | Adj. 3 |  |  |
|  |  | % (N) | OR | 95% CI | p | p | AOR | 95% CI | p | AOR | 95% CI | p |
| Sleep latency | ≥20 min | 12.0% (60) | 2.33 | 1.65-3.29 | <0.001 | <0.001 | 1.87 | 1.29-2.71 | 0.001 | 1.73 | 1.18-2.53 | <0.001 |
|  | <20 min | 5.5% (87) |  |  |  |  |  |  |  |  |  |  |
| Night awakenings | ≥3x/night | 13.0% (33) | 2.22 | 1.47-3.35 | <0.001 | <0.001 | 1.86 | 1.20-2.87 | 0.005 | 1.83 | 1.16-2.86 | 0.009 |
|  | <3x/night | 6.3% (115) |  |  |  |  |  |  |  |  |  |  |
| Early morning awakenings | ≥3x/week | 11.5% (17) | 1.77 | 1.04-3.02 | 0.037 | 0.045 | 1.46 | 0.83-2.57 | 0.194 | 1.38 | 0.77-2.48 | 0.277 |
|  | <3x/week | 6.8% (132) |  |  |  |  |  |  |  |  |  |  |
| Sleep quality | Rather poor/poor | 12.4 % (33) | 2.07 | 1.37-3.11 | 0.001 | 0.001 | 1.50 | 0.96-2.34 | 0.073 | 1.37 | 0.87-2.17 | 0.176 |
|  | Good/not good or bad | 6.4% (116) |  |  |  |  |  |  |  |  |  |  |
| Short sleep | ≤6 h | 14.0% (13) | 2.21 | 1.20-4.07 | 0.011 | 0.014 | 1.32 | 0.68-2.58 | 0.412 | 1.08 | 0.54.2.18 | 0.823 |
|  | >6 h | 6.9% (136) |  |  |  |  |  |  |  |  |  |  |
| Short sleep | ≤7 h | 8.6% (45) | 1.31 | 0.91-1.89 | 0.145 | 0.169 | 1.13 | 0.77-1.66 | 0.539 | 1.10 | 0.75-1.64 | 0.622 |
|  | >7 h | 6.7% (104) |  |  |  |  |  |  |  |  |  |  |
| Insuff. total sleep time | Yes | 14.9% (25) | 2.51 | 1.58-4.01 | <0.001 | <0.001 | 1.86 | 1.13-3.06 | 0.015 | 1.59 | 0.95-2.68 | 0.081 |
|  | No | 6.5% (113) |  |  |  |  |  |  |  |  |  |  |
| Decreased wellbeing | Yes | 16.8% (33) | 3.11 | 2.03-4.75 | <0.001 | <0.001 | 1.93 | 1.21-3.09 | 0.006 | 1.59 | 0.97-2.61 | 0.064 |
|  | No | 6.1% (104) |  |  |  |  |  |  |  |  |  |  |
| Decr. functioning | Yes | 17.0% (27) | 2.99 | 1.90-4.72 | <0.001 | <0.001 | 1.91 | 1.17-3.17 | 0.011 | 1.60 | 0.94-2.71 | 0.082 |
|  | No | 6.4% (111) |  |  |  |  |  |  |  |  |  |  |

Adj. 1 adjusted for background variables (mother’s age when the child was born; primi/multipara; education, three classes; income, three classes; somatic disease/disability

Adj. 2 adjusted for background variables and simultaneous depressive symptoms (EPDS ≥ 11 at T1)

Adj. 3 adjusted for background variables, simultaneous depressive symptoms, history of depression and simultaneous anxiety (SCL ≥10)

b) Insomnia symptoms in middle pregnancy (gw 24, T2) vs EPDS ≥ 11 three months postnatally

|  |  |  | T2 |  |  |  |  |  |  |  |  |  |
| --- | --- | --- | --- | --- | --- | --- | --- | --- | --- | --- | --- | --- |
| Sleep variables at T2 |  | PDS+  3 months | Unadj. |  |  | Adj.1 | Adj.2 |  |  | Adj. 3 |  |  |
|  |  | % (N) | OR | 95% CI | p | p | AOR | 95% CI | p | AOR | 95% CI | p |
| Sleep latency | ≥ 20 min | 10.0% (53) | 1.77 | 1.24-2.52 | 0.002 | 0.007 | 1.26 | 0.85-1.86 | 0.258 | 1.06 | 0.71-1.60 | 0.764 |
|  | <20 min | 5.9% (93) |  |  |  |  |  |  |  |  |  |  |
| Night awakenings | ≥3x/night | 10.3% (31) | 1.70 | 1.12-2.58 | 0.013 | 0.030 | 1.35 | 0.86-2.12 | 0.199 | 1.15 | 0.72-1.86 | 0.558 |
|  | <3x/night | 6.3% (115) |  |  |  |  |  |  |  |  |  |  |
| Early morning awakenings | ≥3x/week | 9.2% (15) | 1.42 | 0.81-2.48 | 0.221 | 0.418 | 0.89 | 0.48-1.66 | 0.714 | 0.801 | 0.42-1.53 | 0.502 |
|  | <3x/week | 6.7% (130) |  |  |  |  |  |  |  |  |  |  |
| Sleep quality | Rather poor/poor | 12.2% (37) | 2.16 | 1.46-3.21 | <0.001 | 0.001 | 1.43 | 0.92-2.22 | 0.115 | 1.20 | 0.76-1.90 | 0.408 |
|  | Good/not good or bad | 6.0% (109) |  |  |  |  |  |  |  |  |  |  |
| Short sleep | ≤6 h | 10.8% (12) | 1.68 | 0.90-3.13 | 0.105 | 0.247 | 0.91 | 0.45-1.83 | 0.781 | 0.87 | 0.43-1.79 | 0.706 |
|  | >6 h | 6.7% (135) |  |  |  |  |  |  |  |  |  |  |
| Short sleep | ≤7 h | 8.9% (54) | 1.49 | 1.05-2.12 | 0.024 | 0.020 | 1.33 | 0.91-1.94 | 0.136 | 1.33 | 0.90-1.95 | 0.148 |
|  | >7 h | 6.2% (93) |  |  |  |  |  |  |  |  |  |  |
| Insuff. total sleep time | Yes | 17.9% (30) | 3.27 | 2.11-5.07 | <0.001 | <0.001 | 1.99 | 1.21-3.28 | 0.007 | 1.66 | 0.99-2.78 | 0.056 |
|  | No | 6.2% (112) |  |  |  |  |  |  |  |  |  |  |
| Decreased wellbeing | Yes | 22.6% (30) | 4.40 | 2.81-6.89 | <0.001 | <0.001 | 2.23 | 1.31-3.79 | 0.003 | 1.86 | 1.08-3.19 | 0.025 |
|  | No | 6.2% (113) |  |  |  |  |  |  |  |  |  |  |
| Decr. functioning | Yes | 17.9% (31) | 3.25 | 2.11-5.01 | <0.001 | <0.001 | 2.02 | 1.24-3.29 | 0.005 | 1.64 | 0.99-2.71 | 0.054 |
|  | No | 6.3% (112) |  |  |  |  |  |  |  |  |  |  |

Adj. 1 adjusted for background variables (mother’s age when the child was born; primi/multipara; education, three classes; income, three classes; somatic disease/disability

Adj. 2 adjusted for background variables and simultaneous depressive symptoms (EPDS ≥ 11 at T2)

Adj. 3 adjusted for background variables, simultaneous depressive symptoms, history of depression and simultaneous anxiety (SCL ≥10)

c) Insomnia symptoms in late pregnancy (gw 34, T3) vs EPDS ≥ 11 three months postnatally.

|  |  |  | T3 |  |  |  |  |  |  |  |  |  |
| --- | --- | --- | --- | --- | --- | --- | --- | --- | --- | --- | --- | --- |
| Sleep variables at T3 |  | PDS+ | Unadj. |  |  | Adj.1 | Adj.2 |  |  | Adj. 3 |  |  |
|  |  | % (N) | OR | 95% CI | p | p | AOR | 95% CI | p | AOR | 95% CI | p |
| Sleep latency | ≥ 20 min | 10.4% (73) | 2.28 | 1.61-3.23 | <0.001 | <0.001 | 1.73 | 1.18-2.55 | 0.005 | 1.59 | 1.07-2.35 | 0.022 |
|  | <20 min | 4.8% (65) |  |  |  |  |  |  |  |  |  |  |
| Night awakenings | ≥3x/night | 8.7% (61) | 1.51 | 1.07-2.13 | 0.019 | 0.025 | 1.20 | 0.81-1.73 | 0.347 | 1.14 | 0.77-1.68 | 0.520 |
|  | <3x/night | 6.0% (82) |  |  |  |  |  |  |  |  |  |  |
| Early morning awakenings | ≥3x/week | 12.9% (32) | 2.29 | 1.51-3.49 | <0.001 | <0.001 | 1.54 | 0.95-2.51 | 0.079 | 1.50 | 0.92-2.45 | 0.106 |
|  | <3x/week | 6.1% (110) |  |  |  |  |  |  |  |  |  |  |
| Sleep quality | Rather poor/poor | 12.4% (75) | 2.93 | 2.08-4.14 | <0.001 | <0.001 | 2.15 | 1.45-3.18 | <0.001 | 2.07 | 1.39-3.08 | <0.001 |
|  | Good/not good or bad | 4.6% (67) |  |  |  |  |  |  |  |  |  |  |
| Short sleep | ≤6 h | 10.4% (19) | 1.66 | 0.96-2.63 | 0.051 | 0.034 | 1.33 | 0.76-2.34 | 0.314 | 1.20 | 0.68-2.14 | 0.531 |
|  | >6 h | 6.5% (122) |  |  |  |  |  |  |  |  |  |  |
| Short sleep | ≤7 h | 9.7% (55) | 1.75 | 1.23-2.49 | 0.002 | 0.001 | 1.74 | 1.18-2.57 | 0.006 | 1.68 | 1.13-2.50 | 0.011 |
|  | >7 h | 5.8% (86) |  |  |  |  |  |  |  |  |  |  |
| Insuff. total sleep time | Yes | 18.7% (41) | 3.97 | 2.68-5.90 | <0.001 | <0.001 | 2.76 | 1.73-4.40 | <0.001 | 2.51 | 1.57-4.03 | <0.001 |
|  | No | 5.5% (99) |  |  |  |  |  |  |  |  |  |  |
| Decreased wellbeing | Yes | 16.7% (31) | 3.18 | 2.07-4.90 | <0.001 | <0.001 | 1.50 | 0.90-2.50 | 0.123 | 1.30 | 0.77-2.19 | 0.328 |
|  | No | 5.9% (109) |  |  |  |  |  |  |  |  |  |  |
| Decr. functioning | Yes | 13.6% (36) | 2.52 | 1.68-3.77 | <0.001 | <0.001 | 1.52 | 0.96-2.40 | 0.075 | 1.35 | 0.84-2.16 | 0.215 |
|  | No | 5.9% (104) |  |  |  |  |  |  |  |  |  |  |

Adj. 1 adjusted for background variables (mother’s age when the child was born; primi/multipara; education, three classes; income, three classes; somatic disease/disability

Adj. 2 adjusted for background variables and simultaneous depressive symptoms (EPDS ≥ 11 at T3)

Adj. 3 adjusted for background variables, simultaneous depressive symptoms, history of depression and simultaneous anxiety (SCL ≥10)
