# Supplementary material for: Estimating the cumulative risk of postnatal depressive symptoms: the role of insomnia symptoms across pregnancy
Source: Soc Psychiatry Psychiatr Epidemiol. 2021 May 7;56(12):2251–61. doi: 10.1007/s00127-021-02101-0 (PMC8558280; doi:10.1007/s00127-021-02101-0)
Supplement: Supplementary file 2 — Supplementary file2 (DOCX 27 KB) [file 127_2021_2101_MOESM2_ESM.docx]

**Online Resource 2.** Predictive model for PDS (EPDS without sleep item ≥ 11, three months postnatally), N = 1504 (complete case data)

1. Best models for time points T2 and T3

|  | T2 best model |  |  | T3 best model |  |  |
| --- | --- | --- | --- | --- | --- | --- |
| Variable | Beta (SE) | p | AIC/BIC | Beta (SE | P | AIC/BIC |
| Intercept | -3.979 (0.327) | 2E^-16^ | **751.095/** 777.674 | -5.270 (0.414) | 2E^-16^ | **732.739/** 759.318 |
| Decr. func. T2 | 0.830 (0.270) | 0.002 |  | - | - |  |
| Subj. insuff. sleep T2 | 0.446 (0.291) | 0.125 |  | - | - |  |
| Anxiety SCL≥10 T2 | 0.726 (0.279 | 0.009 |  | - | - |  |
| EPDS≥11 T2 | 1.024 (0.279 | <0.001 |  | - | - |  |
| Sleep latency >20min T3 | - | - |  | 0.508 (0.210) | 0.015 |  |
| Insuff. sleep T3 | - | - |  | 0.618 (0.269) | 0.022 |  |
| Decr. wellbeing T3 | - | - |  | 0.433 (0.289) | 0.134 |  |
| EPDS≥11 T3 | - | - |  | 1.578 (0.243) | <0.001 |  |

1. Best reduced model for T1, combined model T1+T2 (background variables + T1+T2 measures) and T1+T2+T3 (all available information)

| EPDS without sleep item | T1  Best model |  |  | T1+T2  Best model |  |  | T1+T2+T3  Best model |  |  |
| --- | --- | --- | --- | --- | --- | --- | --- | --- | --- |
| Variable | Beta (std error) | p | AIC/BIC | Beta (std error) | P | AIC/BIC | Beta (std error) | p | AIC/BIC |
| Intercept | -4.856 (0.500) | <2E^-16^ | **738.560/** 786.403 | -5.409 (0.545) | 2E^-16^ | **723.088/** 781.563 | -5.941 (0.534) | 2E^-16^ | **703.175/** 761.650 |
| Parity (primi vs multi) | 0.603 (0.212) | 0.004 |  | 0.493 (0.214) | 0.021 |  | 0.348 (0.224) | 0.121 |  |
| Income level 1 | 0.226 (0.256) | 0.377 |  | 0.144 (0.259) | 0.579 |  | 0.100 (0.261) | 0.701 |  |
| Income level 2 | -0.380 (0.317) | 0.232 |  | -0.427 (0.323) | 0.186 |  | -0.434 (0.326) | 0.183 |  |
| History of depression | 0.862 (0.246) | <0.001 |  | 0.770 (0.250) | 0.002 |  | 0.765 (0.251) | 0.002 |  |
| Sleep latency $\geq$20min T1 | 0.515 (0.219 | 0.019 |  | 0.500 (0.223) | 0.025 |  | 0.476 (0.225) | 0.035 |  |
| Decreased funct. T1 | 0.809 (0.274) | 0.003 |  | 0.608 (0.289) | 0.036 |  | 0.646 (0.290) | 0.026 |  |
| Anxiety SCL ≥10 T1 | 0.518 (0.336) | 0.124 |  | - | - |  | - | - |  |
| EPDS ≥11 T1 | 0.857 (0.298) | 0.004 |  | 0.670 (0.281) | 0.017 |  | - | - |  |
| Decreased funct. T2 | - | - |  | 0.656 (0.280) | 0.019 |  | 0.631 (0.285) | 0.027 |  |
| Anxiety SCL ≥10 T2 | - | - |  | 0.425 (0.295) | 0.149 |  | - | - |  |
| EPDS ≥11 T2 | - | - |  | 0.732 (0.301) | 0.015 |  | 0.666 (0.279) | 0.017 |  |
| Insuff. sleep T3 | - | - |  | - | - |  | 0.508 (0.265) | 0.055 |  |
| EPDS ≥11 T3 | - | - |  | - | - |  | 1.270 (0.259) | <0.001 |  |

EPDS= The Edinburgh Postnatal Depression scale; SCL= The anxiety subscale of Symptom Checklist 90
