# Supplementary material for: Estimating the cumulative risk of postnatal depressive symptoms: the role of insomnia symptoms across pregnancy
Source: Soc Psychiatry Psychiatr Epidemiol. 2021 May 7;56(12):2251–61. doi: 10.1007/s00127-021-02101-0 (PMC8558280; doi:10.1007/s00127-021-02101-0)
Supplement: Supplementary file 3 — Supplementary file3 (DOCX 36 KB) [file 127_2021_2101_MOESM3_ESM.docx]

Online Resource 3.

Table A. Linear regression analysis, insomnia symptoms in early pregnancy (gw 14, T1) vs EPDS ≥ 11 three months postnatally

| Prenatal sleeping problems |  | Crude |  |  |  | AOR^a^ |  |  |  | AOR^b^ |  |  |  |
| --- | --- | --- | --- | --- | --- | --- | --- | --- | --- | --- | --- | --- | --- |
|  |  | Unstand B | SE | stand B | p | Unstand B | SE | stand B | p | Unstand B | SE | stand B | p |
| Sleep latency | ≥ 20min | 1.145 | 0.195 | 0.128 | <0.001 | 1.059 | 0.197 | 0.119 | <0.001 | 0.728 | 0.192 | 0.082 | <0.001 |
|  | <20min |  |  |  |  |  |  |  |  |  |  |  |  |
| Night awakenings | ≥3x/night | 0.807 | 0.256 | 0.069 | 0.002 | 0.748 | 0.260 | 0.064 | 0.004 | 0.452 | 0.251 | 0.039 | 0.072 |
|  | <3x/night |  |  |  |  |  |  |  |  |  |  |  |  |
| Early morning awakenings | ≥3x/week | 0.794 | 0.327 | 0.053 | 0.015 | 0.807 | 0.331 | 0.054 | 0.015 | 0.514 | 0.319 | 0.034 | 0.107 |
|  | <3x/week |  |  |  |  |  |  |  |  |  |  |  |  |
| Sleep quality | rather poor/poor | 1.294 | 0.250 | 0.113 | <0.001 | 1.253 | 0.253 | 0.109 | <0.001 | 0.761 | 0.247 | 0.067 | 0.002 |
|  | good/ not good or bad |  |  |  |  |  |  |  |  |  |  |  |  |
| Short sleep | ≤6h | 1.269 | 0.407 | 0.068 | 0.002 | 1.268 | 0.411 | 0.068 | 0.002 | 0.441 | 0.401 | 0.024 | 0.272 |
|  | >6h |  |  |  |  |  |  |  |  |  |  |  |  |
| Short sleep | ≤7h | 0.464 | 0.194 | 0.052 | 0.017 | 0.464 | 0.195 | 0.053 | 0.018 | 0.271 | 0.188 | 0.031 | 0.150 |
|  | >7h |  |  |  |  |  |  |  |  |  |  |  |  |
| Insuff. total sleep time | yes | 1.845 | 0.309 | 0.136 | <0.001 | 1.817 | 0.310 | 0.134 | <0.001 | 1.382 | 0.301 | 0.102 | <0.001 |
|  | no |  |  |  |  |  |  |  |  |  |  |  |  |
| Decreased wellbeing | yes | 1.945 | 0.287 | 0.154 | <0.001 | 1.923 | 0.287 | 0.153 | <0.001 | 1.073 | 0.288 | 0.086 | <0.001 |
|  | no |  |  |  |  |  |  |  |  |  |  |  |  |
| Decr functioning | yes | 2.028 | 0.316 | 0.146 | <0.001 | 2.008 | 0.317 | 0.144 | <0.001 | 1.158 | 0.316 | 0.084 | <0.001 |
|  | no |  |  |  |  |  |  |  |  |  |  |  |  |

Adj. 1 adjusted for background variables (mother’s age when the child was born; primi/multipara; education, three classes; income, three classes; somatic disease/disability

Adj. 2 adjusted for background variables and simultaneous depressive symptoms (EPDS ≥11 at T1)

Table B. Linear regression analysis, insomnia symptoms in middle pregnancy (gw 24, T2) vs EPDS ≥ 11 three months postnatally

| Prenatal sleeping problems |  | Crude |  |  |  | AOR^a^ |  |  |  | AOR^b^ |  |  |  |
| --- | --- | --- | --- | --- | --- | --- | --- | --- | --- | --- | --- | --- | --- |
|  |  | Unstand B | SE | stand B | p | Unstand B | SE | stand B | p | Unstand B | SE | stand B | p |
| Sleep latency | ≥ 20min | 1.202 | 0.190 | 0.137 | <0.001 | 1.106 | 0.198 | 0.125 | <0.001 | 0.755 | 0.193 | 0.086 | <0.001 |
|  | <20min |  |  |  |  |  |  |  |  |  |  |  |  |
| Night awakenings | ≥3x/night | 0.463 | 0.237 | 0.042 | 0.052 | 0.296 | 0.247 | 0.027 | 0.231 | 0.047 | 0.239 | 0.004 | 0.844 |
|  | <3x/night |  |  |  |  |  |  |  |  |  |  |  |  |
| Early morning awakenings | ≥3x/week | 1.119 | 0.311 | 0.078 | <0.001 | 1.000 | 0.316 | 0.071 | 0.002 | 0.558 | 0.307 | 0.039 | 0.070 |
|  | <3x/week |  |  |  |  |  |  |  |  |  |  |  |  |
| Sleep quality | rather poor/poor | 1.152 | 0.236 | 0.106 | <0.001 | 1.091 | 0.246 | 0.100 | <0.001 | 0.570 | 0.241 | 0.052 | 0.018 |
|  | good/ not good or bad |  |  |  |  |  |  |  |  |  |  |  |  |
| Short sleep | ≤6h | 1.396 | 0.372 | 0.081 | <0.001 | 1.374 | 0.386 | 0.080 | <0.001 | 0.707 | 0.376 | 0.041 | 0.060 |
|  | >6h |  |  |  |  |  |  |  |  |  |  |  |  |
| Short sleep | ≤7h | 0.706 | 0.184 | 0.083 | <0.001 | 0.776 | 0.190 | 0.092 | <0.001 | 0.573 | 0.184 | 0.068 | 0.002 |
|  | >7h |  |  |  |  |  |  |  |  |  |  |  |  |
| Insuff. total sleep time | yes | 2.178 | 0.306 | 0.158 | <0.001 | 2.110 | 0.322 | 0.152 | <0.001 | 1.418 | 0.316 | 0.102 | <0.001 |
|  | no |  |  |  |  |  |  |  |  |  |  |  |  |
| Decreased wellbeing | yes | 2.578 | 0.341 | 0.169 | <0.001 | 2.480 | 0.359 | 0.160 | <0.001 | 1.271 | 0.365 | 0.082 | 0.001 |
|  | no |  |  |  |  |  |  |  |  |  |  |  |  |
| Decr functioning | yes | 1.890 | 0.304 | 0.139 | <0.001 | 1.792 | 0.317 | 0.130 | <0.001 | 0.860 | 0.318 | 0.063 | 0.007 |
|  | no |  |  |  |  |  |  |  |  |  |  |  |  |

Adj. 1 adjusted for background variables (mother’s age when the child was born; primi/multipara; education, three classes; income, three classes; somatic disease/disability

Adj. 2 adjusted for background variables and simultaneous depressive symptoms (EPDS ≥11 at T2)

Table C. Linear regression analysis, insomnia symptoms in late pregnancy (gw 34, T3) vs EPDS ≥ 11 three months postnatally

| Prenatal sleeping problems |  | Crude |  |  |  | AOR^a^ |  |  |  | AOR^b^ |  |  |  |
| --- | --- | --- | --- | --- | --- | --- | --- | --- | --- | --- | --- | --- | --- |
|  |  | Unstand B | SE | stand B | p | Unstand B | SE | stand B | p | Unstand B | SE | stand B | p |
| Sleep latency | ≥ 20min | 1.230 | 0.174 | 0.155 | <0.001 | 0.454 | 0.182 | 0.060 | 0.013 | 0.770 | 0.173 | 0.097 | <0.001 |
|  | <20min |  |  |  |  |  |  |  |  |  |  |  |  |
| Night awakenings | ≥3x/night | 0.570 | 0.176 | 0.071 | 0.001 | 0.509 | 0.181 | 0.064 | 0.005 | 0.262 | 0.172 | 0.033 | 0.128 |
|  | <3x/night |  |  |  |  |  |  |  |  |  |  |  |  |
| Early morning awakenings | ≥3x/week | 1.196 | 0.255 | 0.103 | <0.001 | 1.059 | 0.266 | 0.090 | <0.001 | 0.586 | 0.255 | 0.050 | 0.022 |
|  | <3x/week |  |  |  |  |  |  |  |  |  |  |  |  |
| Sleep quality | rather poor/poor | 1.609 | 0.180 | 0.193 | <0.001 | 1.524 | 0.188 | 0.184 | <0.001 | 1.082 | 0.181 | 0.131 | <0.001 |
|  | good/ not good or bad |  |  |  |  |  |  |  |  |  |  |  |  |
| Short sleep | ≤6h | 0.734 | 0.293 | 0.055 | 0.012 | 0.744 | 0.303 | 0.056 | 0.014 | 0.381 | 0.287 | 0.029 | 0.185 |
|  | >6h |  |  |  |  |  |  |  |  |  |  |  |  |
| Short sleep | ≤7h | 0.749 | 0.186 | 0.088 | <0.001 | 0.767 | 0.193 | 0.091 | <0.001 | 0.621 | 0.183 | 0.074 | 0.001 |
|  | >7h |  |  |  |  |  |  |  |  |  |  |  |  |
| Insuff. total sleep time | yes | 2.589 | 0.266 | 0.212 | <0.001 | 2.576 | 0.278 | 0.212 | <0.001 | 1.875 | 0.269 | 0.155 | <0.001 |
|  | no |  |  |  |  |  |  |  |  |  |  |  |  |
| Decreased wellbeing | yes | 2.220 | 0.288 | 0.169 | <0.001 | 2.213 | 0.300 | 0.167 | <0.001 | 1.033 | 0.300 | 0.078 | 0.001 |
|  | no |  |  |  |  |  |  |  |  |  |  |  |  |
| Decr functioning | yes | 1.822 | 0.247 | 0.162 | <0.001 | 1.694 | 0.255 | 0.151 | <0.001 | 0.973 | 0.248 | 0.087 | <0.001 |
|  | no |  |  |  |  |  |  |  |  |  |  |  |  |

Adj. 1 adjusted for background variables (mother’s age when the child was born; primi/multipara; education, three classes; income, three classes; somatic disease/disability

Adj. 2 adjusted for background variables and simultaneous depressive symptoms (EPDS ≥11 at T3)
