# Supplementary material for: Estimating the cumulative risk of postnatal depressive symptoms: the role of insomnia symptoms across pregnancy
Source: Soc Psychiatry Psychiatr Epidemiol. 2021 May 7;56(12):2251–61. doi: 10.1007/s00127-021-02101-0 (PMC8558280; doi:10.1007/s00127-021-02101-0)
Supplement: Supplementary file 4 — Supplementary file4 (DOCX 16 KB) [file 127_2021_2101_MOESM4_ESM.docx]

Online resource 4 Descriptive statistics for insomnia symptoms at each time point.

|  |  | T1 | T2 | T3 |
| --- | --- | --- | --- | --- |
| Sleep variables |  | % (N) | % (N) | % (N) |
| Sleep latency | ≥20 min | 24.1% (506) | 25.2% (539) | 34.5% (714) |
|  | <20 min | 75.9% (1590) | 74.8% (1598) | 65.5% (1356) |
| Night awakenings | ≥3x/night | 12.2% (257) | 14.2% (303) | 34.0% (707) |
|  | <3x/night | 87.8% (1842) | 85.8% (1833) | 66% (1373) |
| Early morning awakenings | ≥3x/week | 7.1% (150) | 7.8% (166) | 12.2% (253) |
|  | <3x/week | 92.9% (1950) | 92.2% (1971) | 87.8% (1828) |
| Sleep quality | Rather poor/poor | 12.8% (270) | 14.4% (309) | 29.3% (611) |
|  | Good/not good or bad | 87.2 (1835) | 85.6% (1830) | 70.7% (1473) |
| Short sleep | ≤6 h | 4.5% (95) | 5.3% (113) | 8.9% (185) |
|  | >6 h | 95.5% (2004) | 94.7% (2023) | 91.1% (1891) |
| Short sleep | ≤7 h | 25.2¤ (529) | 28.6% (611) | 27.7% (575) |
|  | >7 h | 74.8% (1570) | 71.4% (1525) | 72.3% (1501) |
| Insuff. total sleep time | Yes | 8.8% (169) | 8.5% (170) | 10.7% (220) |
|  | No | 91.2% (1758) | 91.5% (1819) | 89.3% (1827) |
| Decreased wellbeing | Yes | 10.4% (199) | 6.9% (136) | 9.2% (188) |
|  | No | 89.6% (1718) | 93.1% (1840) | 90.8% (1863) |
| Decr. functioning | Yes | 8.5% (162) | 9.0% (177) | 13.2% (271) |
|  | No | 91.5% (1753) | 91.0% (1799) | 86.8% (1786) |
